# Surgical Management of Perforative Peritonitis Following Gastroduodenal Peptic Perforation: A Prospective Observational Study

**DOI:** 10.7759/cureus.101181

**Published:** 2026-01-09

**Authors:** Prabhdeep S Chowdhary, Jaspreet S Bedi, Sujit K Sah

**Affiliations:** 1 General Surgery, Mata Gujri Memorial Medical College and Lions Seva Kendra Hospital, Kishanganj, IND

**Keywords:** duodenal perforation, gastric perforation, graham's patch, peptic ulcer perforation, perforative peritonitis, surgical management

## Abstract

Introduction

Peptic ulcer perforation is a life-threatening surgical emergency with significant morbidity and mortality, particularly in resource-limited settings. This study aimed to evaluate the surgical management and outcomes of patients with perforative peritonitis secondary to gastroduodenal perforation, analyzing demographic patterns, clinical presentations, surgical techniques, and postoperative outcomes.

Methods

A prospective observational study was performed over 18 months at Mata Gujri Memorial Medical College and Lions Seva Kendra Hospital in Kishanganj, Bihar, India. The study enrolled thirty patients who exhibited clinical symptoms and radiological signs consistent with gastroduodenal perforation. Collected data included demographic information, clinical presentation, laboratory results, imaging studies, operative findings, surgical interventions utilized, and postoperative outcomes. The primary surgical methods for repair involved Graham's patch and the modified Graham's patch techniques.

Results

The study population had a mean age of 50.23 ± 18.45 years with male predominance (21/30, 70%, M:F ratio 2.33:1). Duodenal perforations were more common (20/30, 66.67%) than gastric perforations (10/30, 33.33%). All patients presented with abdominal pain, while 15/30 (50%) had nausea or vomiting. Most perforations were small, less than 0.5 cm, observed in 20/30 (66.67%) cases. Graham’s omental patch technique was predominantly used, 16/30 (53.33%) for duodenal and 6/30 (20%) for gastric perforations. Gastric perforations showed higher complication rates, with 2/10 (20%) abscess formation and 1/10 (10%) mortality, compared to duodenal perforations, which had 3/20 (15%) abscess rate and 1/20 (5%) mortality.

Conclusion

Perforative peritonitis due to peptic ulcer disease remains a significant surgical emergency predominantly affecting middle-aged males. Graham's omental patch repair offers favorable outcomes in majority of cases. Gastric perforations demonstrate higher morbidity and mortality compared to duodenal perforations. Prompt diagnosis, timely surgical intervention, and efficient perioperative care are critical for improving clinical outcomes.

## Introduction

Peptic ulcer perforation represents a life-threatening surgical emergency, affecting approximately 2-10% of patients with peptic ulcer disease globally. According to WHO estimates and systematic reviews, the global incidence of peptic ulcer perforation ranges from 3.8 to 10 per 100,000 population annually, with significant geographic variation. Mortality rates range from 1.3% to 20%, making prompt diagnosis and appropriate surgical intervention critical [1-3]. Developed nations report mortality rates of 1.3-5%, while resource-limited settings experience rates of 10-20% due to delayed presentation and limited perioperative care. Despite advances in medical management, perforation remains one of the most catastrophic complications of peptic ulcer disease, demanding immediate surgical attention [4,5]. A recent systematic review by Shahi et al. comprehensively analyzed etiologies and outcomes following duodenal perforation, highlighting the continued clinical significance of this condition [6].

Key risk factors include Helicobacter pylori infection and non-steroidal anti-inflammatory drug (NSAID) use, particularly in elderly populations [7-8]. In India, the epidemiological landscape differs from Western populations, with perforation rates of 5-15 per 100,000 population, higher male subject predominance (male:female subject ratio 7-10:1), and younger presentation age (mean 35-45 years versus 55-65 years in Western countries) [9-11]. In rural Bihar, including Kishanganj district, delayed presentation remains a major challenge due to geographic barriers, limited healthcare infrastructure, and economic constraints, with 40-50% of patients presenting beyond 48 hours compared to 15-20% in urban centers. This delay correlates with higher rates of generalized peritonitis and mortality (15-25% rural versus 5-10% urban) [10].

Graham's omental patch and modified Graham's patch techniques are the most commonly employed surgical approaches for peptic perforations [12-15]. The choice of repair technique depends on perforation size, location, patient stability, and peritonitis severity [16,17], with risk stratification using Mannheim Peritonitis Index (MPI) and Acute Physiology and Chronic Health Evaluation II (APACHE II) scores guiding perioperative decisions [18-20].

Despite the clinical significance of this condition, comprehensive data comparing outcomes between gastric and duodenal perforations remain limited from resource-constrained settings in India. Most existing studies focus on Western populations or urban tertiary centers, with insufficient representation of rural Indian demographics [11]. Limited evidence exists regarding comparative complication profiles and mortality patterns between anatomical perforation sites in this regional context.

This study aimed to evaluate the clinical presentation, surgical management strategies, and postoperative outcomes of patients with gastroduodenal perforation presenting with perforative peritonitis, and to compare morbidity and mortality patterns between gastric and duodenal perforations in a tertiary care center in Kishanganj, Bihar, India.

## Materials and methods

Study design

A prospective observational study was designed to assess the surgical management approaches and postoperative outcomes in patients experiencing perforative peritonitis due to gastroduodenal perforation. The research was carried out at Mata Gujri Memorial Medical College and Lions Seva Kendra Hospital, Kishanganj, Bihar, India. The study duration was 18 months. The study enrolled 30 patients presenting clinical symptoms and radiological indications consistent with gastroduodenal perforation. Sample size was calculated using the formula n = (z² × σ²) / e² with z=1.96 (95% confidence), σ=0.40, and e=0.15, yielding n≈28, rounded to 30 patients.

Study population

The study systematically screened all patients coming to the Casualty or Outpatient Department of the Department of Surgery, who were suspected of gastroduodenal perforation and who fulfilled entry criteria (inclusion and exclusion criteria).

Inclusion criteria

Patients who were operated on within 48 hours of hospital admission and were willing to participate in the study with appropriate informed consent.

Exclusion criteria

Patients were excluded if any of the following conditions were met: history of abdominal trauma to rule out traumatic perforations, Patients under 21 years were excluded as this threshold aligns with institutional protocols distinguishing adult from adolescent surgical populations, and peptic ulcer disease in younger patients may have different etiological profiles requiring separate analysis.

Data Collection

Data collection included demographics, clinical history (symptom duration, peptic ulcer history, NSAID use, smoking and alcohol consumption), physical examination findings, laboratory investigations (complete blood count, electrolytes, renal and liver function tests, C-reactive protein, arterial blood gas), imaging findings (chest X-ray, abdominal ultrasound, contrast-enhanced CT when required), intraoperative findings (perforation site, size, peritoneal contamination, surgical technique), and postoperative outcomes (complications, hospital stay duration, mortality). Disease severity was assessed using the Mannheim Peritonitis Index (MPI) [18] and Acute Physiology and Chronic Health Evaluation II (APACHE II) scores [20].

Surgical management

Preoperative management included fluid resuscitation, nasogastric decompression, and broad-spectrum antibiotics. Surgical repair utilized Graham's patch or modified Graham's patch techniques with comprehensive peritoneal lavage.

Statistical analysis

Data were analyzed using Python 3.10 (Python Software Foundation, Fredericksburg, VA, US), Pandas (open-source data analysis library, Python Software Foundation, Fredericksburg, VA, US), and SciPy Continuous (open-source scientific computing library, SciPy developers) variables were expressed as mean ± standard deviation (SD) and categorical variables as frequencies and percentages. Due to the descriptive observational nature and small sample size (n=30), inferential statistical tests were not performed. Comparisons between gastric and duodenal perforation groups are presented as observed differences without statistical significance testing. This study was conducted and reported in accordance with Strengthening the Reporting of Observational Studies in Epidemiology (STROBE) guidelines for observational studies [21].

Ethical considerations

Institutional ethical approval was obtained from the ethics committee at Mata Gujri Memorial Medical College and Lions Seva Kendra Hospital, Kishanganj, Bihar, prior to study initiation (approval no. MSM/IEC-21/2025). All patients provided voluntary informed consent. Patient confidentiality and data protection protocols were rigorously maintained throughout the study.

## Results

The mean age was 50.23 ± 18.45 years, with the 41-50 years age group being most prevalent (40.0%) (Table 1).

**Table 1:** Age distribution of patients with perforative peritonitis secondary to gastroduodenal perforation (n=30)

Age group	Frequency	Percentage (%)
21-30	4	13.3
31-40	8	26.7
41-50	12	40
>50	6	20

Regarding sex distribution, male subjects represented (21/30, 70%) while female subjects accounted for (9/30, 30%), resulting in a male-to-female subject ratio of 2.33: 1, indicating a significant male gender predominance in the peptic perforation cases.

Comorbidities were present in 56.7% of patients, with hypertension (20%), pulmonary disease (13.3%), and diabetes mellitus (10%) being most common (Table 2).

**Table 2:** Distribution of comorbidities among patients with perforative peritonitis (n=30)

Comorbidity	Frequency	Percentage (%)
Hypertension	6	20
Pulmonary disease (chronic obstructive pulmonary disease (COPD))	4	13.3
Diabetes mellitus	3	10
Chronic kidney disease	2	6.7
Bladder outlet obstruction	1	3.3
Urinary tract infection	1	3.3
Hypothyroidism	1	3.3

All patients presented with abdominal pain (100%) and tenderness (100%). Nausea and vomiting occurred in 50%, while guarding (80%) and rebound tenderness (46.7%) reflected varying degrees of peritoneal irritation.

Laboratory findings demonstrated leukocytosis (White blood cells (WBC): 14,325 ± 5,435 cells/μL), elevated CRP (48.2 ± 22.6 mg/L), and raised lactate dehydrogenase (LDH) (320 ± 112 U/L). Mean MPI and APACHE II scores were 13.85 ± 6.23 and 6.21 ± 7.23, respectively, indicating moderate peritonitis with mild systemic involvement (Table 3).

**Table 3:** Laboratory parameters and severity scores in patients with perforative peritonitis WBC: white blood cells; LDH: raised lactate dehydrogenase (LDH); MPI: Mannheim Peritonitis Index; APACHE II: Acute Physiology and Chronic Health Evaluation II.

Variable	Mean	SD	Reference range
WBC (cells/mL)	14325	5435	4,000-11,000
CRP (mg/L)	48.2	22.6	<10
LDH (U/L)	320	112	140-280
MPI score	13.85	6.23	May-30
APACHE II score	6.21	7.23	0-71

**Figure 1:**
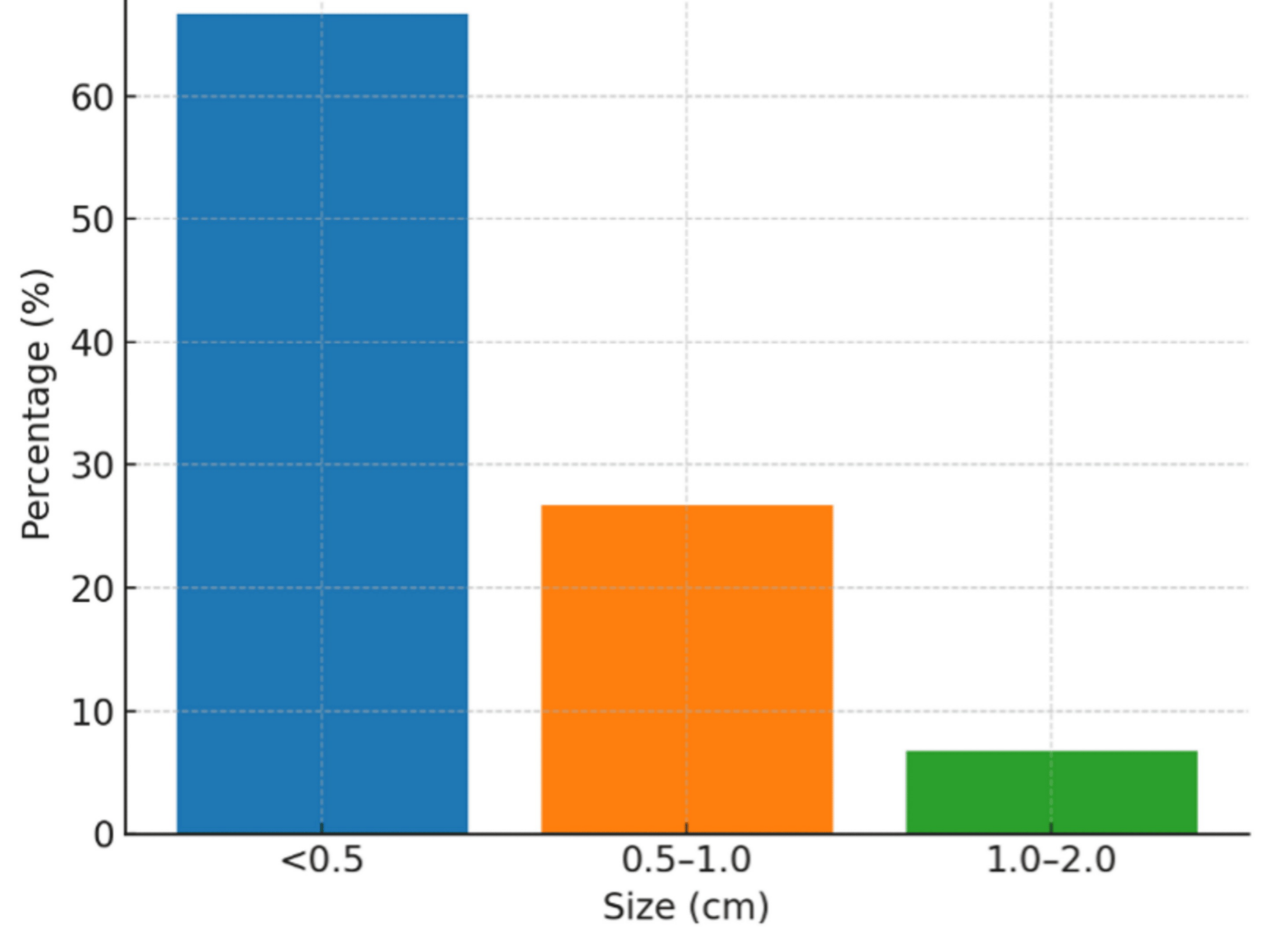
Distribution of perforation sizes Majority of cases (66.7%) involving small perforations measuring less than 0.5 cm in diameter.

Duodenal perforations (66.67%) were more common than gastric perforations (33.33%), with the anterior gastric wall being the predominant gastric site. Most perforations (66.7%) were small (<0.5 cm) (Figure 1).

**Figure 2:**
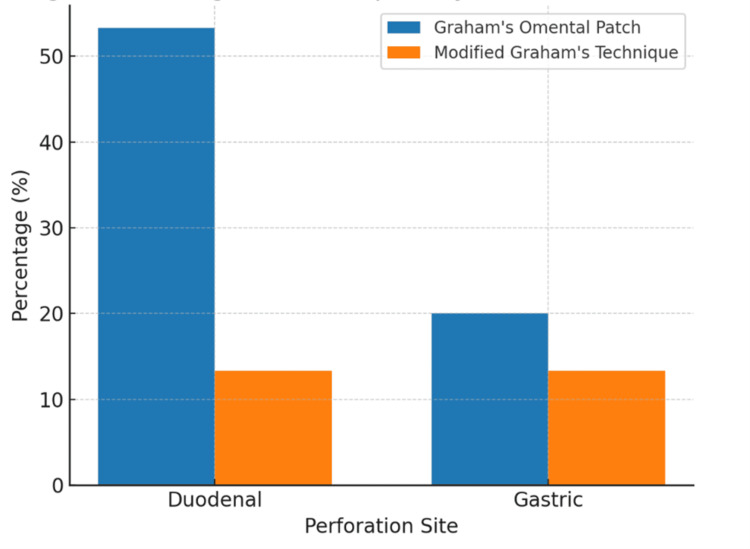
Surgical techniques employed for gastric and duodenal perforations Graham's omental patch was the predominant technique used.

Most procedures (60%) were completed within one to 1.5 hours. Graham's omental patch was the predominant technique for both duodenal (53.3%) and gastric (20%) perforations (Figure 2).

**Figure 3:**
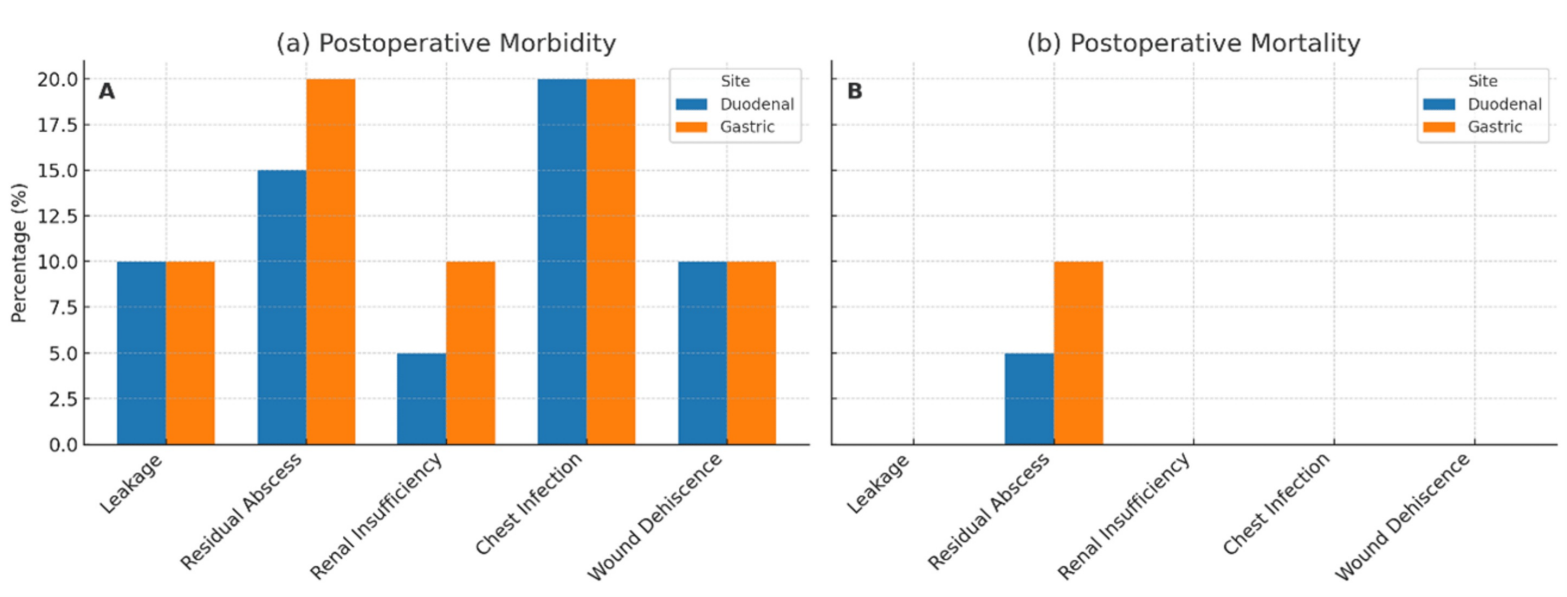
Postoperative complications comparison between duodenal and gastric perforations Higher complication rates were observed in gastric perforations, including increased (A) abscess formation (20% vs 15%) and (B) mortality (10% vs 5%).

Postoperative complications demonstrated significant differences between perforation sites. Duodenal perforations (n=20) showed a 15% residual abscess rate (three cases) with associated 5% mortality (one death), while gastric perforations (n=10) demonstrated higher complication severity with 20% abscess formation (two cases) and 10% mortality (one death). Both sites shared similar rates of leakage (10%), wound dehiscence (10%) rates, and chest infection (20%). All mortality cases resulted from abscess-related sepsis (Figure 3).

Postoperative recovery metrics demonstrated favorable outcomes with mean time to oral intake of 3.2 ± 1.1 days (range two to seven days), average intensive care unit (ICU) stay of 1.5 ± 0.8 days (range zero to five days), and return to work after 4.5 ± 1.2 weeks (range three to eight weeks) (Table 4).

**Table 4:** Postoperative recovery parameters in patients with perforative peritonitis ICU: Intensive care unit.

Parameter	Mean	SD	Range
Time to oral intake (days)	3.2	1.1	2–7
ICU stay (days)	1.5	0.8	0–5
Return to work (weeks)	4.5	1.2	3–8

## Discussion

This study provides insights into the surgical management and outcomes of 30 patients with perforative peritonitis secondary to gastroduodenal perforation, revealing demographic, clinical, and surgical trends that contribute to understanding this surgical emergency.

The predominance of the 41-50 years age group (40%) with a mean age of 50.23 ± 18.45 years mirrors findings reported by previous studies [22], though some research has identified younger demographic peaks, suggesting potential regional or risk-factor variations related to NSAID usage patterns and H. pylori prevalence [11]. The significant male subject predominance (70%, male: female individuals = 2.33:1) aligns consistently with global literature [23,24], likely reflecting higher rates of smoking and alcohol consumption among male subjects, factors known to contribute to peptic ulcer development and complications.

The finding that 56.7% of patients had at least one pre-existing comorbidity, with hypertension being most prevalent (20.0%), followed by pulmonary disease (13.3%) and diabetes mellitus (10%), corresponds with established literature documenting the association between these conditions and peptic ulcer complications [20]. The presence of chronic kidney disease (6.7%), while less frequently reported, represents an important consideration for postoperative outcomes due to its association with delayed wound healing and increased sepsis risk. The universal presence of abdominal pain (100%) underscores its diagnostic significance as the cardinal symptom of peptic ulcer perforation, corroborating multiple previous studies [22,23]. The observation that nausea and vomiting occurred in 50% of cases indicates variability in gastrointestinal symptom manifestation, likely influenced by perforation size and duration of presentation. The relatively lower prevalence of bowel obstruction symptoms (23.3%) suggests variable bowel involvement, potentially correlating with perforation characteristics and timing of medical presentation [25].

Universal tenderness (100%) reinforces its role as the most consistent sign, while guarding (80.0%) and rebound tenderness (46.7%) reflect varying peritoneal irritation severity, consistent with established literature [26].

The marked inflammatory response demonstrated by leukocytosis (14,325 ± 5,435 cells/μL) and elevated CRP (48.2 ± 22.6 mg/L) indicates robust systemic inflammation consistent with secondary peritonitis pathophysiology. The elevation in LDH (320 ± 112 U/L) supports tissue necrosis and cellular injury presence. The moderate peritonitis severity indicated by MPI scores (13.85 ± 6.23) and mild systemic illness reflected by APACHE II scores (6.21 ± 7.23) align with previous research, emphasizing the prognostic value of these scoring systems [27].

The predominance of duodenal perforations (66.67%) over gastric perforations (33.33%) with anterior wall involvement corresponds with anatomical expectations and existing literature [12]. This distribution pattern reflects the anatomical vulnerability of these sites to acidic gastric contents and mechanical stress, supporting established understanding of perforation pathophysiology.

The finding that two-thirds of patients (66.7%) had small perforations (<0.5 cm) while only 6.7% had larger defects (1-2 cm) aligns with previous research, indicating that larger perforations are associated with increased mortality and more complex postoperative courses [20]. This size distribution has important implications for surgical planning and outcome prediction. The predominant use of Graham's omental patch technique, particularly for duodenal perforations (53.3%), with favorable outcomes supports this approach as the gold standard for peptic perforation repair. The operative efficiency demonstrated by 60% of procedures being completed within 1-1.5 hours reflects the technique's reliability and the predominance of smaller perforations in the study population.

The higher complication rates observed in gastric perforations, including doubled mortality risk (10% vs 5%), emphasize the increased clinical severity associated with gastric compared to duodenal perforations. The finding that all mortality cases resulted from abscess-related sepsis highlights the critical importance of source control and postoperative infection surveillance [28]. The identification of abscess formation as the primary cause of mortality underscores the need for aggressive perioperative management and vigilant postoperative monitoring. The favorable recovery parameters, including mean time to oral intake (3.2 ± 1.1 days), short ICU stays (1.5 ± 0.8 days), and reasonable return to work timelines (4.5 ± 1.2 weeks), indicate satisfactory functional outcomes in most patients. These metrics likely reflect the benefits of early surgical intervention, standardized repair techniques, and comprehensive perioperative support protocols [29,30]. These findings align with the systematic review by Shahi et al. which reported variable mortality rates based on perforation etiology and management strategies [6].

The study findings reinforce several important clinical principles. First, prompt recognition and surgical intervention remain paramount for optimal outcomes. Second, while Graham's omental patch technique provides reliable results for most perforations, gastric cases require heightened vigilance due to increased complication risks. Third, comprehensive preoperative risk assessment using validated scoring systems can guide perioperative management decisions. Fourth, aggressive source control and postoperative infection prevention strategies are essential for reducing mortality rates.

This study has several limitations that should be acknowledged. The relatively small sample size of 30 patients limits the statistical power and generalizability of findings. The single-center design may introduce selection bias and limit external validity across different healthcare settings. The 18-month study duration, while adequate for the sample size, may not capture seasonal variations in disease presentation. Additionally, the study lacked a control group comparing different surgical techniques, and long-term follow-up data beyond immediate postoperative recovery were not assessed. The exclusion of pediatric patients and those with severe comorbidities may have resulted in selection bias toward healthier adults, potentially underestimating overall morbidity and mortality rates in the general population with peptic perforations.

The results support the continued use of Graham's omental patch as the primary surgical technique for peptic perforation repair while emphasizing the need for individualized risk assessment and tailored postoperative management strategies based on anatomical location and patient-specific factors.

## Conclusions

Perforative peritonitis secondary to peptic ulcer disease predominantly affects middle-aged males, with duodenal perforations being more common than gastric perforations. Graham's omental patch repair remains an effective surgical technique with favorable outcomes in most cases. Gastric perforations demonstrate higher morbidity and mortality compared to duodenal perforations, with abscess-related sepsis being the primary cause of mortality. Prompt diagnosis, timely surgical intervention, aggressive infection control, and vigilant postoperative monitoring are critical for improving clinical outcomes. Future multicenter studies with larger cohorts are needed to validate these findings and establish evidence-based protocols for resource-constrained settings.
